# A novel non-invasive diagnostic sampling technique for cutaneous leishmaniasis

**DOI:** 10.1371/journal.pntd.0005750

**Published:** 2017-07-13

**Authors:** Yasaman Taslimi, Pardis Sadeghipour, Sima Habibzadeh, Vahid Mashayekhi, Hossien Mortazavi, Ingrid Müller, Majella E. Lane, Pascale Kropf, Sima Rafati

**Affiliations:** 1 Department of Immunotherapy and *Leishmania* Vaccine Research, Pasteur Institute of Iran, Tehran, Iran; 2 Emam Reza Hospital, Mashhad University of Medical Sciences, Mashhad, Iran; 3 Department of Dermatology, Razi Hospital, Tehran University of Medical Sciences, Tehran, Iran; 4 Department of Medicine, Imperial College London, London, United Kingdom; 5 University College London, School of Pharmacy, London, United Kingdom; Institut Pasteur de Tunis, TUNISIA

## Abstract

Accurate diagnosis of cutaneous leishmaniasis (CL) is important for chemotherapy and epidemiological studies. Common approaches for *Leishmania* detection involve the invasive collection of specimens for direct identification of amastigotes by microscopy and the culturing of promastigotes from infected tissues. Although these techniques are highly specific, they require highly skilled health workers and have the inherent risks of all invasive procedures, such as pain and risk of bacterial and fungal super-infection. Therefore, it is essential to reduce discomfort, potential infection and scarring caused by invasive diagnostic approaches especially for children. In this report, we present a novel non-invasive method, that is painless, rapid and user-friendly, using sequential tape strips for sampling and isolation of DNA from the surface of active and healed skin lesions of CL patients. A total of 119 patients suspected of suffering from cutaneous leishmaniasis with different clinical manifestations were recruited and samples were collected both from their lesions and from uninfected areas. In addition, 15 fungal-infected lesions and 54 areas of healthy skin were examined. The duration of sampling is short (less than one minute) and species identification by PCR is highly specific and sensitive. The sequential tape stripping sampling method is a sensitive, non-invasive and cost-effective alternative to traditional diagnostic assays and it is suitable for field studies as well as for use in health care centers.

## Author summary

Cutaneous leishmaniasis is highly endemic in many countries and definitive diagnosis of disease is crucial. Accurate diagnosis of cutaneous leishmaniasis requires isolation of infected tissues, culturing of parasites from infected lesion and microscopic identification of parasites. Both approaches are invasive and cause discomfort especially for children. Here, we described a novel method using sequential tape strips for sampling and isolation of DNA from the surface of skin lesions of CL patients for specific diagnosis. This method is a sensitive, non-invasive and cost-effective alternative to traditional diagnostic assays. Furthermore, it is painless, rapid, user-friendly and suitable for field studies and heath care centers.

## Introduction

The leishmaniases are a complex of neglected tropical diseases affecting more than 12 million people in 98 countries, with 350 million people at risk [1]. The clinical status of the disease can be classified as localized cutaneous, mucocutaneous, diffuse and visceral leishmaniasis. Different species of *Leishmania* are transmitted to the human host by the bite of infected female sandfly vectors. Cutaneous leishmaniasis is endemic in 87 countries and caused by various species of protozoan parasites of the genus *Leishmania* such as *L*. *major*, *L*. *tropica*, *L*. *infantum*, *L*. *aethiopica*, *L*. *mexicana*, *L*. *amazonensis* and *L*. *braziliensis*. It is the most significant protozoan infection in India, Central, South, and Western Asian countries as well as in countries surrounding the Mediterranean Sea [2]. The annual incidence of CL is between 0.7 and 1.2 million cases worldwide. The incidence may change from year to year and the actual number of cases may not be accurate due to underreporting. There is no satisfactory treatment for any of the different forms of CL [3]. Available treatments include pentavalent antimonials, sodium stibogluconate (Pentostam, Stibanate) and meglumineantimoniate (Glucantime), which have been used for over half a century [4].

Cutaneous leishmaniasis starts as a papule at the site of the sandfly bite and is usually associated with cell infiltration (induration), followed by ulceration and eventually re-epithelialization and scarring. Usually lesions heal in more than 90% of cases in a time span of 3–18 months [5]. The symptoms of leishmaniasis can vary and may be confused with skin diseases caused by other etiological agents; therefore diagnostic confirmation of the parasite is mandatory for starting the specific treatment. For diagnosis of CL, parasitological detection is essential and it is usually based on direct detection of amastigotes in smears from lesions by microscopy and by the culturing of promastigotes from infected tissues [6–8]. Although these techniques are highly specific, they are time consuming, require skilled health workers and are not able to identify the causative strain of *Leishmania*. The ability to type the infecting *Leishmania* species is important for treatment and control measures as well for epidemiological studies. A recent approach using polymerase chain reaction (PCR)-based methods for species identification is a specific, sensitive and reliable alternative tool as compared to traditional techniques [9–11]. Some available invasive techniques such as punch biopsies, scrapings and aspirates of ulcers are among the most commonly used clinical specimen collection methods for diagnosis of CL [12–15]. Besides requiring technical expertise, these invasive approaches carry the risk of bleeding, pain as well as bacterial and fungal super-infections. Therefore, non-invasive, painless, safer, simpler and more sensitive diagnostic procedures are urgently required, especially for children.

In this study, we described the use of tape strip sampling as a non-invasive method for diagnosis and isolation of DNA from the surface of infected and non-infected areas of the skin of CL patients. By using this non-invasive method, species identification can be performed in different stages of the disease including acute, chronic and healed lesions. Therefore, the use of the non-invasive sampling technique described here will facilitate diagnosis of CL and identification of the infecting *Leishmania* species.

## Methods

### Ethics statement

The present study was approved by the Ethics Committee of the Pasteur Institute of Iran and the Razi Skin Hospital affiliated with the Tehran University of Medical Sciences. All sampling from CL patients was performed under the supervision of dermatologists at Razi Hospital and the Emam Reza Hospital (Mashhad). Fungal skin sampling was carried out in the Mycology Department at the Pasteur Institute of Iran. Sampling from the skin of healthy controls was performed in different locations in Tehran and Mashhad. Sample collection from all the different subjects was carried out by two well-trained research assistants in order to allow for a comparable, uniform sampling approach. All participants signed the informed consent form before sample collection. For children, the written informed consent form was signed by parents or legal guardians on behalf of their children.

### Patients and clinical samples

Patients from different health care centers were referred to the Skin Center of Razi Hospital (Tehran) and the Emam Reza Hospital (Mashhad) for diagnosis of CL. Patients with clinical appearance of erythematous nodules or ulcerated lesions with a minimal size of 1 cm in diameter and a duration of more than one month were selected by a dermatologist. Positive diagnosis of CL (n = 119) was based on the microscopic detection of *Leishmania* parasites in Giemsa-stained lesion smears. The duration of the disease was defined as starting from the time the patients noticed the appearance of skin inflammation and lesions and the clinical and parasitological confirmation of CL. Lesions were sampled from a total of 119 referred patients; 49 were female, 70 were male and the median age was 30 years (average 33±20.27, range: minimum 1/maximum 79 years old) as shown in Table 1. Specimens were obtained from the nose, lips, ears, eyelids, chin, cheeks, neck, legs, arms, and fingers of the patients. It is worth to mention that the sizes of lesion are different in different patients. Furthermore, 15 cases with fungal infections of the skin were also included in this study. As negative controls, samples were collected from the skin of 54 non-infected healthy individuals without previous history of CL.

**Table 1:** Clinical profiles of 119 cutaneous leishmaniasis patients.

Profiles	
• Age (year)^a^	33±20.27
• Gender	
Male	70(58%)
Female	49(42%)
• Average number of lesion	2.7±3.3
• Lesion site on	
Lip	6(5%)
Eyelid	2(1.6%)
Ear	2(1.6%)
Other sites of face	32(26.6%)
Hand	40(33.3%)
Leg	17(14.1%)
Mix	21(17.5%)

### Direct examination

The touch smear of dermal tissue of each patient was prepared by superficial incision on the margin of the lesion against a glass microscope slide. After the smear had dried completely, it was fixed with absolute methanol and stained with Giemsa for microscopic examination for presence of amastigote.

### Study design

As a pilot study, thirty-one confirmed active CL cases were selected in order to compare the sensitivity of two different approaches. For the invasive sampling approach, 2 mm biopsies were collected from the border of ulcerated lesion using a sterile disposable punch. Each biopsy was used to culture under the sterile condition for parasite propagation. For non-invasive skin sampling, one tape disc (D-Squame, CuDerm Corporation, Texas, USA) with a diameter of 22 mm was placed on an infected and uninfected area. For further validation, eighty-eight new confirmed CL patients were diagnosed through non-invasive sampling.

### Parasite culture

The fragmented biopsy from the border of the ulcers were collected and inoculated into Schneider’s Drosophila medium (Sigma, Darmstadt, Germany), supplemented with 10% heat- inactivated fetal bovine serum (FBS, Gibco, UK), 40 mM HEPES, 2 mM, 0.1 mM adenosine, 2 mM L-glutamine, 0.5μg/ml hemin (all from Sigma, Germany) and 50 μg/ml gentamicin (Biosera, France) and incubated at 26°C. Cultures were checked microscopically for live and motile promastigotes for maximum of 3–4 weeks.

### Tape strip sampling

For non-invasive skin sampling, one tape disc was placed on selected areas (infected and non-infected areas). In order to apply even pressure to the disc, a plunger was gently held on the selected areas and pressed for approximately 20 seconds as already reported [16,17]. The tape disc was detached and transferred into a sterile 1.5ml vial and stored at 4°C until further use. This sampling is painless for patients, especially for children, and can be used to sample lesions on sensitive parts such as eyelids and lips.

### Isolation and DNA preparation from tape strip discs

In order to isolate the DNA from the tape disc, each strip was divided into eight pie-slice pieces using sterile scissors and placed into a 1.5ml sterile vial. DNA samples were extracted using a slightly modified DNAeasy Blood & Tissue kit (QIAGEN, Germany). To each sample tube 180μl of ATL BQ and 20μl proteinase K were added, gently mixed and incubated for 3 hours at 56°C in a dry block. During incubation the mixture was gently vortexed every 30 minutes. The rest of the steps were done according to the QIAGEN manual. DNA was eluted with 70μl H_2_O and stored at 4°C. The DNA concentration of each extraction was measured using a NanoDrop ND 1000 (5ng-35ng/μl). Genomic DNA from reference strains, *L*. *major* (MHRO/IR/75/ER) and *L*. *tropica* (MOHM/IR/09/Khamesipour-Mashhad) were prepared following the manufacturer’s instructions (QIAGEN, Germany).

### PCR amplification and identification of parasite species

Two PCR assays were used in our experiments: First PCR was targeting the kDNA1 minicircle of *Leishmania* species [18] and the second was for species identification (*L*. *tropica* and *L*. *major)*.

Amplification of kDNA1 minicircle for detection of conserved targets of *Leishmania* species was performed by using a pair of primers F: GGGTAGGGGCGTTCTGC R: TACACCAACCCCCAGTTTGC (Invitrogen). PCR mixtures contained 4mM MgCl_2_, 0.5mM dNTP, 1 Unit of Taq DNA polymerase (Roche, Germany) and 40ng of each primer in a final volume of 50μl. Amplification conditions in an eppendorf thermocycler (Germany) were as follows: 95°C for 5min, 62°C for 1min, 72°C for 1min, 95°C for 45 sec, 65°C for 30 sec, 72°C for 30 sec (30 cycle), 72°C for 5 min. Amplification products were subjected to electrophoresis in 1% agarose in 0.5 X TBE (0.045M Tris-borate, 1mM EDTA) buffer. DNA purified from cultured promastigotes of *L*. *major* and *L*. *tropica* as reference strains was amplified in each PCR experiment as positive control. As negative controls we used water instead of DNA and amplified it in each PCR experiment.

For species identification, the internal transcribed space 1 (ITS1) PCR assay was used with primers LITSR (5´CTGGATCATTTTCCGATG3´) and L5.8S (5´ TGATACCACTTATCGCACTT 3´) to amplify the ribosomal ITS1 region [19,20]. The DNA amplification was performed in a 50μl reaction comprised of 0.5mM dNTP, 1.5mM MgCl_2_, 1U of Taq polymerase (Roche, Germany), 25pmol of each primer and 10ng of DNA from each reference strain or 100-200ng DNA isolated from clinical samples and healthy controls. The cycling conditions were 94°C for 5min, followed by 35 cycles of 94°C for 30sec, 53°C for 1min, and 72°C for 1min and final extension of 72°C for 15min. Then the PCR products were subjected to RFLP analysis by digesting the amplified PCR product with 1U *Hae*III enzyme (Roche) at 37°C for 2 hours. The restriction fragments were analyzed on 2% agarose gels.

### Statistical analysis

Sensitivity comparisons of the sampling techniques used were performed on samples from 31 patients using biopsies, parasite culturing and the non-invasive tape strip disc sampling. The sensitivity of each method was calculated based on the percentage of positivity of the total analyzed samples with 95% confidence interval for each approach. Level of significance was set at *p*<0.05.

## Results

### Sampling and clinical profile of patients

**Fig 1:**
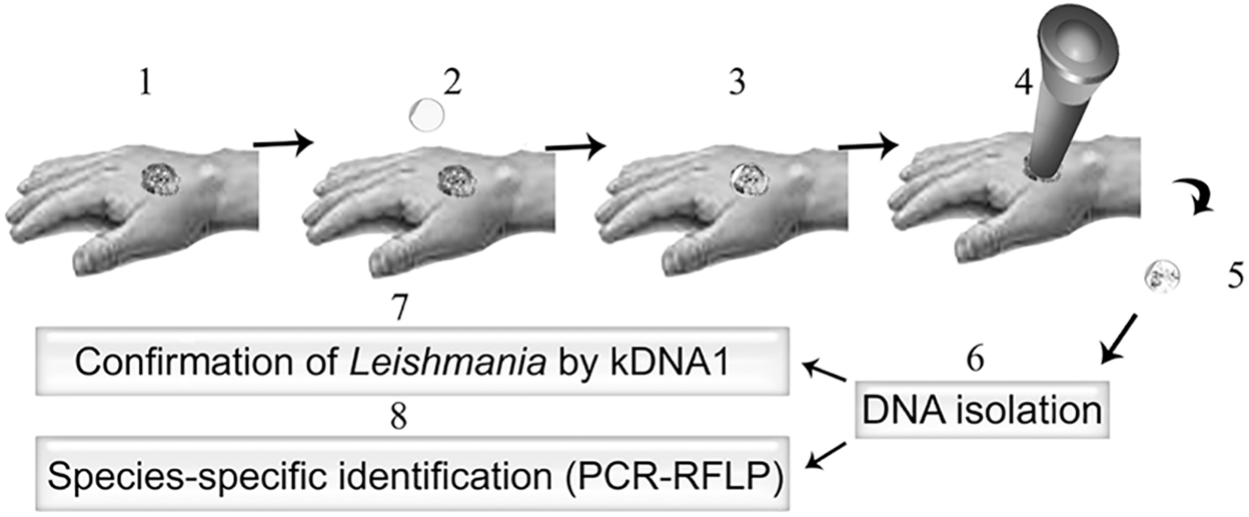
Schematic presentation of sampling lesions with tape strip discs, DNA isolation and further identification of *Leishmania* species. 1) patient´s lesion 2) disc 3) disc applied to patient´s lesion 4) even pressure applied on lesion using a plunger 5) sterile removal of disc from the lesion 6) DNA isolation from disc 7) confirmation of *Leishmania* by kDNA1 8) species-specific identification (PCR ITS-RFLP).

**Fig 2:**
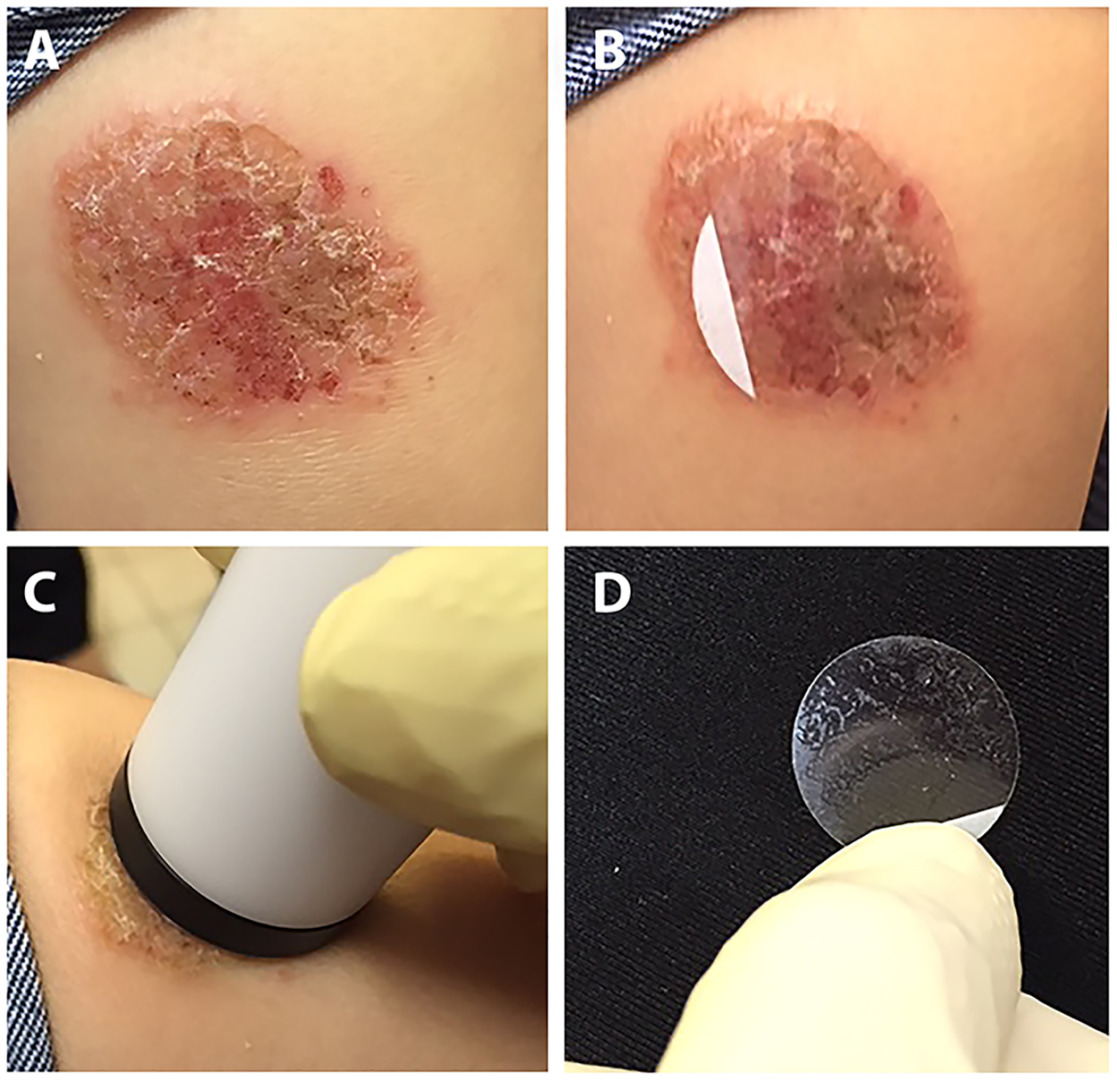
Illustration of sample collection using tape strip disc. A) determine the lesion for disc sampling B) apply the tape strip disc on the lesion C) apply even pressure for 20 seconds with the plunger D) detach the disc and transfer into a sterile vial for DNA isolation.

**Fig 3:**
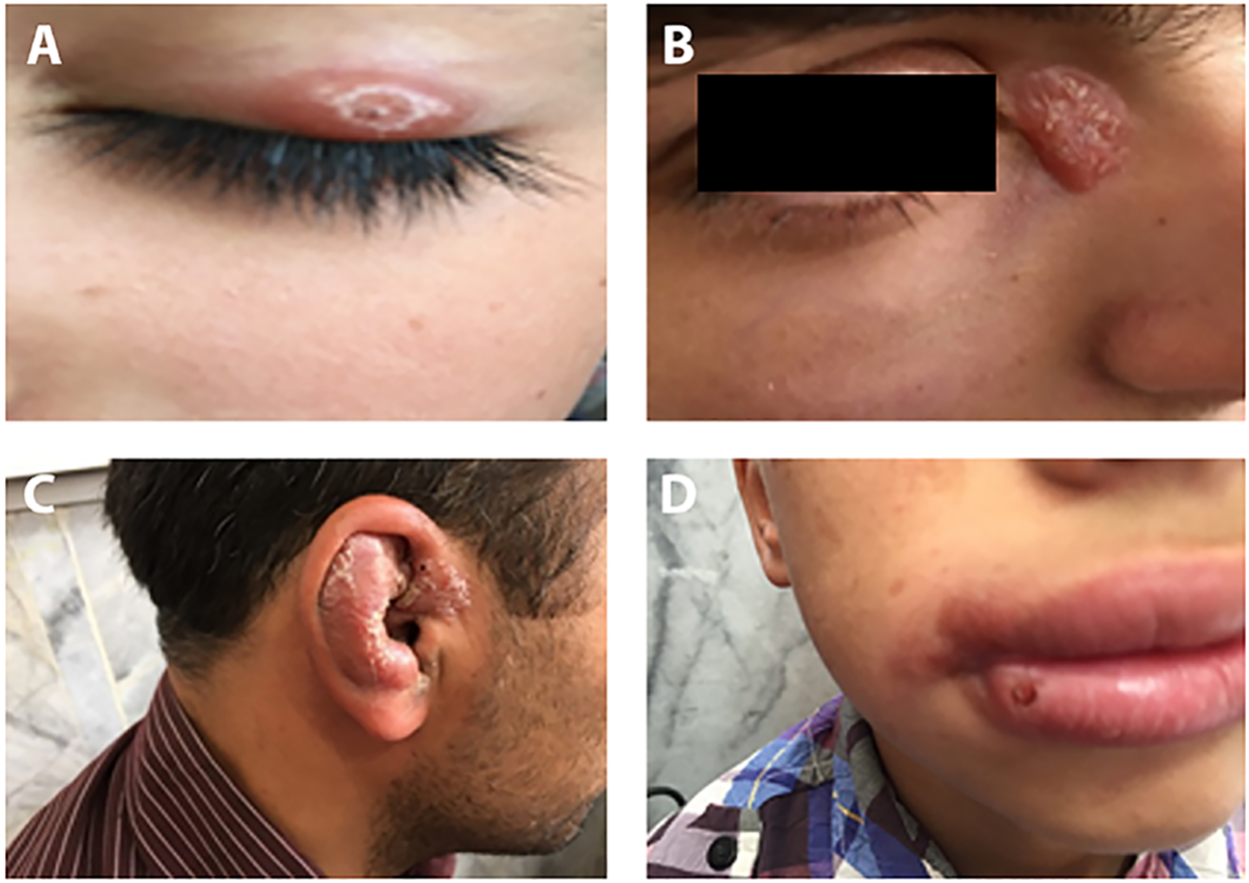
Sensitive areas for sampling. A) eyelid B) around the eye C) ear D) on the lips.

In this study we used a tape stripping technique to sample the affected skin. As shown in Figs 1 and 2, skin sampling can be done in less than a minute and does not need to be performed by highly trained individuals. The method we used is child-friendly, causes virtually no pain, and is very practical for those lesions which occur in sensitive areas such as the eyelids, lips and ears, as shown in Fig 3. Of the 119 confirmed CL patients enrolled in this study, 79 patients (66%) presented with acute CL (duration ranged from 1 up to 12 months), 32 (27%) with chronic CL (duration ranged from 30 up to 360 months) and 8 (7%) with healed CL (recovered for more than 6 months, 70 males and 49 females). The disease duration, gender distribution and infecting species of *Leishmania* are shown in Table 2. In total, 71 patients were infected with *L*. *tropica* (60%), 41 with *L*. *major* (34%) and 7 were undetermined (6%). In our project, 35% of the patients suffered from multiple lesions (more than 3) and the rest had only one lesion as shown in Table 1.

**Table 2:** Disease duration, gender distribution and infecting species of *Leishmania* in CL patients with acute, chronic or healed disease.

	Female	Male	Mean duration in month (min-max)	*% L*. *tropica* infected	*% L*. *major* Infected	Unknown species
Acute CL N = 79	31 (39%)	48 (61%)	5.8 (1–12)	45 (57%)	30 (38%)	4 (5%)
Chronic CL N = 32	15 (47%)	17 (53%)	75.8 (30–360)	18 (56.4%)	11 (34.3%)	3 (9.3%)
Healed CL N = 8	3 (37%)	5 (63%)	9.4 (6–12)	7 (87%)	1 (13%)	0
Total N = 119	49 (42%)	70 (58%)		71 (60%)	41 (34%)	7 (6%)

### Recovery of DNA from skin lesion and non-infected skin using noninvasive stripping disc

**Fig 4:**
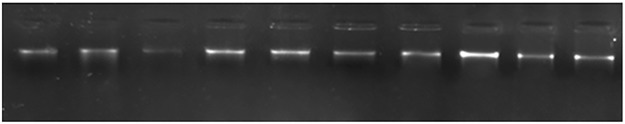
Genomic DNA isolation from healthy and infected skin. The concentration of DNA from healthy skin is lower (lanes 1–3, mean 1.9 ng/μl) as compared to infected skin (lanes 4–10, mean 19.5 ng/μl)

In this study, we collected tape strip samples from both skin lesions and non-infected control sites. Substantially more DNA was harvested from lesions as compared to control skin; an average of 19.5 ng/μl (range: 7 to 34 ng/μl) of DNA was recovered from lesions and an average of 1.9 ng/μl (range: 0.7 to 2.7 ng/μl) was isolated from the control skin sites as shown in Fig 4.

### PCR and identification of *Leishmania* species collected using noninvasive tape strip discs

**Fig 5:**
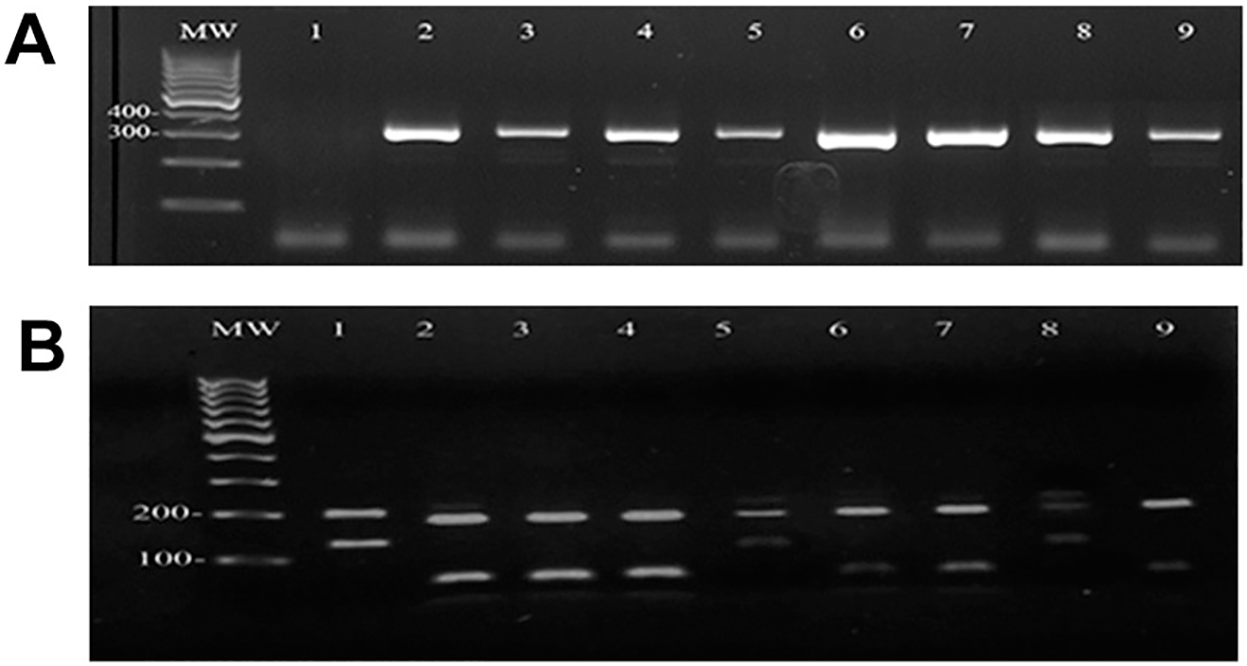
Agarose gel electrophoresis of amplified ITS1 regions of standard reference strains. H_2_O (lane 1) *L*. *major* (lane 2) and *L*. *tropica* (lane 3) and from CL patients (lanes 4–9) in panel A and restriction endonuclease *Hae*III digestion is shown in panel B. In both panels, MW representative of 100-bp ladder as molecular size marker. Patients in samples 2, 3, 4, 6, 7 and 9 are infected with *L*. *tropica* and patients in samples 1, 5 and 8 are infected with *L*. *major*.

In the first step, DNA isolated from all the collected tape strip disc samples, including CL- patients with active, chronic or healed disease, fungal-infected patients and healthy skin, were amplified using kDNA1 minicircle primers. The kDNA1 amplification was positive for all CL patients and yielded 100% sensitivity as shown in Fig 5A. In contrast, none of the samples from patients infected with fungi nor those from healthy skin were positive using the kDNA1 minicircle primers. Standard reference strains of *L*. *major* and *L*. *tropica* were used as positive controls. In the second step, all isolated DNA with positive amplification using kDNA1 were subjected to ITS1 PCR-RFLP for species identification. Standard strains of *L*. *major* and *L*. *tropica* were used as controls. PCR to amplify the ITS1 region gave an amplified fragment of 300bp for all samples from CL patients. Subsequent digestion with the restriction enzyme *Hae*III revealed two bands for *L*. *major* (160 and 210bp) and *L*. *tropica* (50 and 190bp) Fig 5B. Among the 79 acute cases, 45 samples (57%) were diagnosed as *L*. *tropica*, 30 samples (38%) as *L*. *major* and 4 cases (5%) were not amplified with ITS1 primers, as shown in Table 2. From 32 chronic CL cases, 18 samples (56.4%) were diagnosed as *L*. *tropica*, 11 cases (34.3%) as *L*. *major* and 3 samples (9.3%) were not amplified with ITS1 primers. Among the 8 healed samples, 7 samples were diagnosed as *L*. *tropica* and one sample as *L*. *major*. Using ITS1 primers, a total of 112 samples were diagnosed as either *L*. *major* or *L*.*tropica* and showed 94% sensitivity.

### Diagnosis using the non-invasive tape strip disc technique is more sensitive than parasite culture

We compared the sensitivity of the two different diagnostic techniques based on either culturing parasites from the biopsies obtained from 31 confirmed CL patients or based on the non-invasive tape strip disc technique. As shown in Table 3, the diagnostic accuracy of each method was calculated separately as compared to microscopic identification of amastigotes in lesion smears as a diagnostic reference standard. The highest diagnostic sensitivity of 100% (95% CI) was achieved with the tape strip disc technique. Of 31 suspected patients, only 16 were positive for culturing parasite from lesion biopsies (sensitivity 51%) which was significantly lower than tape strip disc sampling (*p*<0.05).

**Table 3:** Diagnostic accuracy of parasite culture from biopsies and tape strip disc.

Sample Type*	TP	FP	FN	TN	Sensitivity (95% CI)
**Biopsy Culture**	16	0	15	0	51% (33% - 69%)
**Tape Strip Disc**	31	0	0	0	100%

*Compared with the reference microscopy diagnostic test (TP, true positive; FP, false positive; FN, false negative; TN, true negative; CI, confidence interval)

## Discussion

Here we present a non-invasive technique for sampling different clinical stages of CL lesions including those from patients with acute, chronic and healed lesions. Tape strip disc sampling is non-invasive, easy to perform, and well tolerated by patients compared to traditional diagnostic methods such as biopsy, aspiration and scraping. In countries where leishmaniasis is highly endemic, the rate of infected children is high and, sometimes due to fear, it is not possible to take samples for diagnostic and follow-up purposes. This alternative, non-invasive sampling technique is patient-friendly and was acceptable to all children involved in this project. The presented non-invasive approach also eliminates the risk of scarring, and co-infections and reduces the costs associated with biopsy, infection or wound care. By using this novel approach for CL sampling, we were able to bypass several intermediary steps and eliminate the need for anesthetic, needles, syringes and sharp biohazard disposal. Many other techniques, such as aspirating and scraping the CL lesion, are highly operator dependent requiring a trained clinician/health care worker for sampling and are difficult to perform routinely in remote endemic areas. The tape strip disc sampling technique is applicable for use in remote endemic areas and permits the easy transfer of the disc to a larger laboratory or reference center for evaluation. The time for sample collection is less than a minute, which is very important for field studies where large-scale sampling is done. Furthermore, the non-invasive tape strip disc sampling could act as an attractive alternative not only for diagnosis of CL but also for monitoring the response of patients to treatment. Recently, Suarez et al. reported a higher parasite load in samples collected using cytology brushes and dermal scrapings as compared to biopsies [21]. This can be explained by the fact that *Leishmania* amastigotes are not homogenously distributed in skin lesions and a higher abundance of parasites are in the upper layer of the dermis. In addition, in a skin punch biopsy, the ratio of human host DNA to parasite DNA is several fold higher compared to methods of scraping and using cytology brushes. These two factors can decrease the sensitivity of detection of the pathogen in clinical samples.

Patients with different confirmed clinical stages of CL and different duration of skin lesions as well as sizes (small to large lesions) and types (ulcerated, non ulcerated, dry, wet) were recruited into this study and our results show that the novel sampling technique we used is very sensitive; we not only detected parasites in tape strip discs in the acute stage of CL, where most CL patients have ulcerative lesions with a high degree of parasite load, we also obtained similar sensitivity in CL patients presenting with chronic lesions that harbour a lower parasite load. The specificity of culturing parasites from biopsies is very high almost 100% but the sensitivity is insufficient. By comparing the sensitivity of the invasive method of parasite culturing from biopsies with non-invasive tape disc in limited number of active CL cases (n = 31), we revealed significantly lower sensitivity for the invasive biopsy based technique (*p*<0.05). There are different factors that may influence the sensitivity of culturing parasites from biopsies including the strain and viability of collected parasites, the media (different requirement for different species), the presence of a super infection and the expertise of the investigator. Different success rates have been reported mostly between 40–50%. There are some reports showing that swabs and cytology brush samples of CL are more sensitive than lesion aspirate and biopsy samples [22]. Furthermore, the combination of swabs with quantitative PCR (qPCR) is emerging as powerful diagnostic tools due to its noninvasive and simple collection method [23].

Although we did not quantify the parasite load in the lesions of CL patients diagnosed by the use of the noninvasive tape strip disc technique, it will be feasible to do this in future studies since the total amount of recovered DNA is sufficient to be used with more precise methods, such as quantitative real time PCR. Therefore, this non-invasive sampling method could not only be used to improve diagnosis of CL, but also to quantify parasite burden in lesions of CL patients. As reported by Prina et al [24], the *Leishmania* DNA detected by PCR is derived from intact parasites and a positive PCR result was correlated to the presence of living parasites. There are other studies that show positive PCR results correspond to viable parasites in infections caused by other protozoan parasites such as *Plasmodium chabaudi*, and *Toxoplasma gondii* [25,26].

In the present study, we easily obtained enough DNA material from 119 CL patients, 15 fungal lesions as well as 54 samples of normal skin for PCR performance using different sets of primers to confirm the *Leishmania* infection and then identify the species in individuals with both acute and chronic disease. Furthermore, we confirmed, in a limited number of healed individuals, the *Leishmania* species. This may require further investigation for both New and Old world leishmaniasis in different countries but promises to open new perspectives for non-invasive diagnosis and epidemiological surveys.
